# Influence of 3D Printer Type, Resin Material, Thickness, and Geometry on the Mechanical Properties of Directly Printed Clear Aligners

**DOI:** 10.3390/polym18121486

**Published:** 2026-06-13

**Authors:** Fırat Oğuz, Sabahattin Bor, Buse Çebi Gül, Handan Göze Oğuz

**Affiliations:** 1 Department of Orthodontics, Faculty of Dentistry, İnönü University, Malatya 44280, Türkiye; venaroshan@gmail.com; 2 Department of Prosthodontics, Faculty of Dentistry, İnönü University, Malatya 44280, Türkiye; buse.cebi@hotmail.com; 3 Private Practice, Malatya 44280, Türkiye; aydin_handan_01@hotmail.com

**Keywords:** clear aligner, 3D printing, direct printing resin, tensile strength, elastic modulus, orthodontic materials

## Abstract

To evaluate the effects of three different 3D printers, two clear aligner resins, two specimen thicknesses, two lengths, and two geometric designs on the tensile strength and elastic modulus of directly printed clear aligners. Specimens were produced from two orthodontic aligner resins, Clear A (Senertek, Izmir, Turkey) and Tera Harz TA 28 (Graphy Inc., Seoul, Republic of Korea), using three different 3D printers: Ackuretta SOL (LCD), Asiga MAX (DLP), and UNIZ NBEE (LCD). Specimens were designed in two forms (dumbbell, in accordance with ISO 527 3, and flat strip), in two thicknesses (0.5 mm and 1 mm), and in two lengths (short and long), yielding 24 groups with 5 specimens each (*n* = 120). All specimens were post processed using the Tera Harz Spinner and cured for 25 min under nitrogen atmosphere in the THC 2 MC unit, followed by a 1 min boiling water treatment. Tensile tests were performed on a universal testing machine (Shimadzu Corp., Kyoto, Japan) up to fracture. Maximum force (N) and elastic modulus (N/mm^2^) were recorded. Data were analyzed using Kruskal–Wallis, Mann–Whitney U, and Aligned Rank Transform ANOVA tests with Dunn post hoc and Bonferroni correction (*p* < 0.05). Printer type had no significant effect on maximum force (*p* = 0.357) or elastic modulus (*p* = 0.052). Resin type (*p* < 0.001), thickness (*p* < 0.001), and specimen geometry (*p* < 0.001) showed significant effects on both parameters. TA 28 specimens exhibited higher mechanical performance than Clear A. Increased thickness produced higher maximum force and elastic modulus values. Flat geometries showed the highest maximum force, while the short dumbbell exhibited the lowest. The long thin dumbbell geometry yielded the highest elastic modulus values. Resin composition, thickness, and specimen geometry are the primary determinants of mechanical performance in directly printed clear aligners, whereas printer type appears to play a limited role.

## 1. Introduction

Approximately 75% of adult patients are dissatisfied with their dental appearance [1]. Consequently, interest in orthodontic treatments aimed at improving esthetic appearance is steadily increasing, and these treatments are being preferred more frequently. This rising demand has also elevated expectations for orthodontic appliances offering superior functional and esthetic properties. Indeed, recent studies have reported that patients seeking orthodontic treatment consider esthetics a primary priority [2,3] and that adult patients embrace aligner treatment more readily than conventional brackets [4].

Clear aligners have emerged as a treatment option capable of providing this esthetic benefit, owing to their removable nature and nearly invisible structure [5]. Thanks to these features, they exhibit fewer adverse effects, such as caries, calculus, or white spot formation, than those caused by traditional fixed appliances [6]. In addition, they offer advantages such as causing less pain in the early days of treatment compared with fixed orthodontic appliances, enabling faster treatment in a shorter period, reducing the number and duration of appointments, and requiring fewer emergency visits [7,8]. On the other hand, clear aligners also have disadvantages, including high production costs, dependence of treatment success on patient cooperation, and the inability to treat certain malocclusions [5,9]. To overcome these disadvantages, recent technological developments have focused particularly on production processes, and the improvement of digital workflows in the manufacture of clear aligners has become an important area of research.

Today, the fact that the world’s leading aligner manufacturers have reached very high daily production capacities has intensified production processes and clearly demonstrated the need for a more efficient, accessible, and error free production workflow [10]. At this point, in-house production and direct 3D printing technologies stand out as remarkable alternatives in the field of orthodontic treatment. With the widespread adoption of 3D printing technologies, in house production has become increasingly feasible, providing clinicians with the ability to produce custom orthodontic appliances, including clear aligners, directly in the clinical setting [11,12,13]. Thus, the role of the clinician in design and production processes is expanding, dependence on aligner companies is decreasing, and rapid replanning becomes possible in the event of errors. On the other hand, the conventional thermoforming workflow that can be performed in-house involves a multi-step manufacturing process, including model printing, thermoforming, and trimming procedures, and has been reported to adversely affect the mechanical properties of aligner materials [14]. In contrast, direct 3D printing eliminates the intermediate thermoforming stages and provides a more standardized manufacturing process. Furthermore, direct 3D printing offers several advantages, including a lower risk of cumulative errors, improved precision, greater control over aligner thickness, and reduced production costs [15,16,17,18]. Owing to these advantages, in house production offers a more efficient clinical workflow compared with traditional production processes that depend on external companies [13,19].

On the other hand, in in-house production, the ultimate clinical efficacy is largely determined by the mechanical properties of the material used [20]. It has been reported that the performance of the thermoplastic material, together with thickness and geometry, determines the effectiveness of orthodontic movements [21]. Therefore, differences in mechanical properties may directly affect the biomechanical success of treatment [2,21]. However, although current knowledge emphasizes the importance of mechanical behavior, it has been observed that the literature has not sufficiently elucidated the effects of 3D printers, different resins, aligner thickness, and length on mechanical performance in aligner production.

Current studies have predominantly focused on the printing quality and dimensional accuracy of different 3D printing technologies. Among these technologies, stereolithography (SLA), digital light processing (DLP), and liquid crystal display (LCD) systems are the most commonly used methods in dental applications [22]. While SLA printing is more frequently employed in research settings and laboratories, DLP and LCD technologies are more widely used for in-house manufacturing. All three technologies are based on the photopolymerization of liquid resins [23]. DLP systems cure an entire layer simultaneously using a projector-based light source. In contrast, LCD printers achieve selective polymerization by allowing light to pass through specific regions of a liquid crystal panel [24]. Although each of these three major 3D printing technologies has its own advantages and limitations, previous studies have generally reported that clinically acceptable and applicable dental models can be produced using all of them [25]. In particular, LCD and DLP technologies have gained increasing popularity in recent years for in-house aligner manufacturing due to their lower cost and faster production capabilities. Nevertheless, the effects of these technologies on the mechanical performance of directly printed aligners have not yet been fully elucidated.

In light of all this information, the determining role of the structural and mechanical differences in the thermoplastic material used in clinical outcomes clearly emerges. The aim of this study is to evaluate the effects of specimens with different thicknesses, lengths, and geometries, obtained from two different clear aligner materials produced with three different 3D printers, on tensile strength and modulus of elasticity. The null hypothesis of the study is that material type, specimen geometry, thickness, length, and the type of 3D printer used have no statistically significant effect on tensile strength and modulus of elasticity.

## 2. Materials and Methods

This study is an experimental laboratory investigation conducted to compare the physical properties of different clear aligner materials. All tests were performed under standard ambient conditions by the same operators.

**Figure 1:**
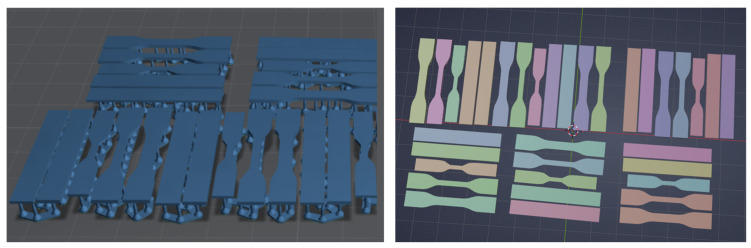
The samples were arranged in Blender after design and viewed with support in a 3D slicer.

The tensile strength and elastic modulus of two different orthodontic aligner materials Clear A (Şenertek, İzmir, Türkiye) and Graphy (Graphy Inc., Seoul, Republic of Korea) were compared. TA-28 and Clear-A resins are among the promising alternative materials that have recently been widely investigated for the fabrication of directly printed clear aligners and have been proposed as effective options for orthodontic treatment [26,27]. Therefore, these two commercially available resins, representing different material formulations used in direct aligner manufacturing, were selected to investigate the effect of resin composition on the mechanical properties of directly printed aligners. Specimens were prepared using standardized designs in both dumbbell (ISO 527-3) [28] and flat-strip forms (Figure 1). In addition, for each form, variations were produced in two different thicknesses (0.5 mm and 1 mm) and two different lengths (short and long).

All specimens were modeled digitally and printed using Ackuretta SOL (Ackuretta, Taipei, Taiwan), Asiga MAX (Asiga, Sydney, Australia), and Uniz NBEE (UNIZ Technology, San Diego, CA, USA) 3D printers (Table 1). Printing was performed using the manufacturer-recommended Graphy printing profiles available for each printer platform. Following fabrication, the specimens were cleaned and post-cured according to the recommended post-processing protocols. No modifications were made to the default printing parameters.

These printers were selected because they represent commercially available LCD and DLP photopolymerization technologies that are commonly used in dental additive manufacturing and frequently reported in the literature [29,30]. Including both technologies allowed the assessment of the potential influence of printing mechanisms on the mechanical properties of directly printed aligner materials.

According to the a priori power analysis performed with G*Power (version 3.1; Heinrich Heine University of Düsseldorf, Düsseldorf, Germany), when the effect size was set at f = 0.5, the significance level at α = 0.05, and the power (1-β) at 0.80, the calculation for 24 groups indicated that the required total sample size was 120 [31]. Accordingly, 5 specimens were used for each subgroup.

After printing, the specimens were carefully removed from the build platform. Uncured resin was eliminated using the Tera Harz Spinner system (Graphy Inc., Seoul, Republic of Korea) for 5 min, employing a combination of centrifuge-based cleaning and internal-environment heating in accordance with the Tera Harz TA-28 instructions for use. Following the spinning process, specimens were air-dried at room temperature prior to post-curing. Post-curing was performed in strict accordance with the manufacturer’s standard protocol, applied identically to both resins. Both Tera Harz TA-28 and Clear-A specimens were cured for 25 min in a nitrogen atmosphere using the THC-2 MC curing unit (Graphy Inc., Seoul, Republic of Korea). Finally, all specimens were treated with boiling water for 1 min.

**Figure 2:**
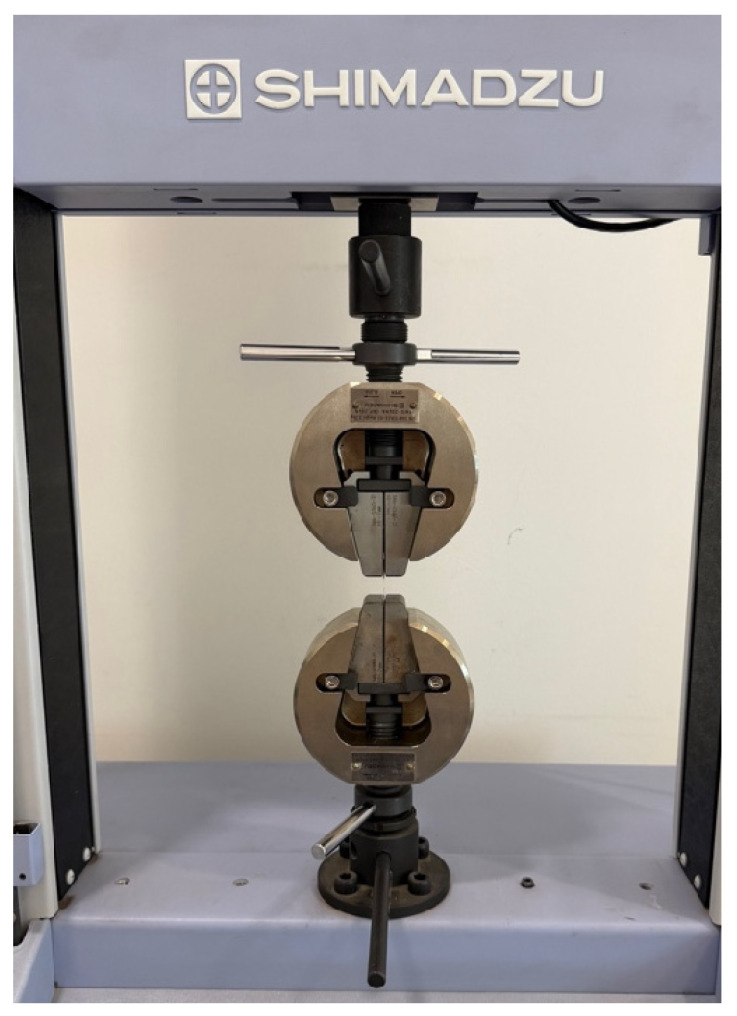
Universal testing machine used for the tensile tests.

Tensile tests for all prepared combinations were performed on a universal testing machine (Shimadzu Corp., Kyoto, Japan) (Figure 2) up to the fracture point under zero preload and at a constant test speed. For each specimen, mechanical data including the maximum tensile strength (N/mm^2^) and modulus of elasticity were recorded in Excel (Microsoft Office 365, Redmond, WA, USA).

### Statistical Analysis

The distribution characteristics of the data were examined using the Shapiro–Wilk normality test, while the homogeneity of variances was assessed with Levene’s test. Since the assumptions of normality and homogeneity were not satisfied across all groups, non-parametric methods were predominantly preferred for comparisons; Kruskal–Wallis and Mann–Whitney U tests were applied for group comparisons, and Dunn’s post hoc test with Bonferroni correction was used to identify significant differences. The Aligned Rank Transform (ART) ANOVA method was preferred for the analysis of multifactorial structures and interactions, while relationships between variables were evaluated using Spearman’s rank correlation. Furthermore, to ensure that the findings were not limited solely to statistical significance, effect sizes were reported using ε^2^ for the Kruskal–Wallis test and the rank-biserial correlation coefficient (r) for the Mann–Whitney U test. The level of statistical significance was set at *p* < 0.05 for all analyses. All statistical analyses were performed using R software (version 4.5.3; R Foundation for Statistical Computing, Vienna, Austria), with the ARTool package employed for Aligned Rank Transform ANOVA.

## 3. Results

Descriptive statistics for the maximum force (N) and elastic modulus (N/mm^2^) values of all experimental groups are presented in Supplementary Tables S1 and S2. In addition, detailed interactions among printer type, resin, and specimen geometry are illustrated as heatmaps in Supplementary Figures S1 and S2. Among all groups, the highest mean maximum force was observed in the UNIZ NBEE (LCD) + TA-28 group (1 mm, flat geometry) at 300.43 N, whereas the lowest value was recorded in the Ackuretta SOL (LCD) + Clear-A group (0.5 mm, short dumbbell geometry) at 14.92 N. In terms of elastic modulus, the highest mean value was obtained in the Asiga MAX (DLP) + TA-28 group (0.5 mm, long thin dumbbell geometry) at 1501.57 N/mm^2^, while the lowest value was found in the Asiga MAX (DLP) + TA-28 group (0.5 mm, short dumbbell geometry) at 9.35 N/mm^2^.

**Figure 3:**
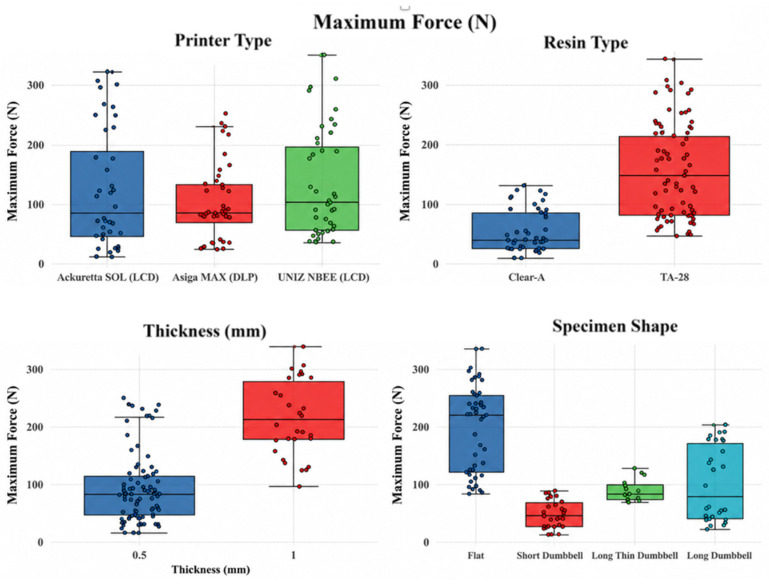
Distribution of maximum force (N) values according to printer type, resin type, specimen thickness, and geometry.

**Figure 4:**
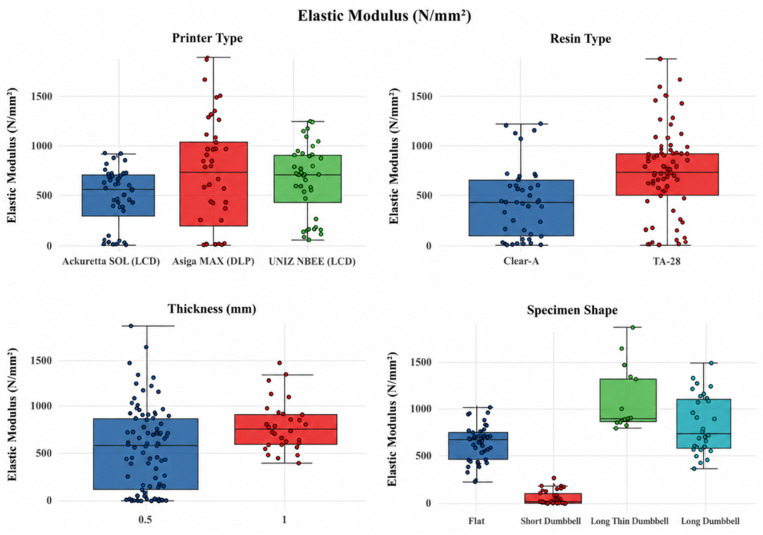
Distribution of elastic modulus (N/mm^2^) values according to printer type, resin type, specimen thickness, and geometry.

**Figure 5:**
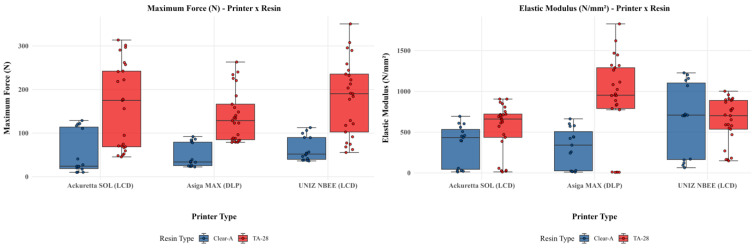
Distribution of maximum force (N) and elastic modulus (N/mm^2^) according to printer type and resin type.

**Figure 6:**
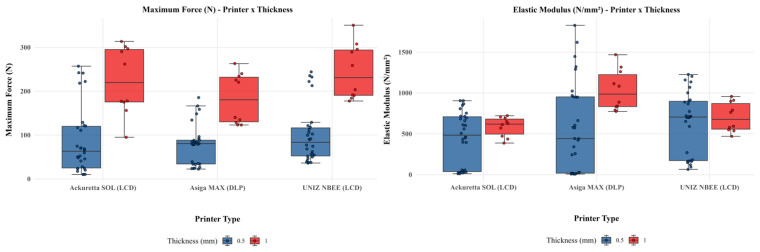
Distribution of maximum force (N) and elastic modulus (N/mm^2^) according to printer type and thickness type.

**Figure 7:**
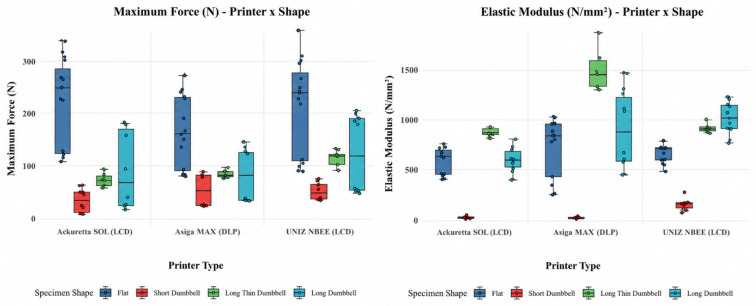
Distribution of maximum force (N) and elastic modulus (N/mm^2^) according to printer type and shape type.

When the classifications based on the main factors are examined in Figure 3, Figure 4, Figure 5, Figure 6 and Figure 7, it is evident that maximum force and elastic modulus values differed markedly among the groups. With respect to printer type, no clear superiority was observed among the groups; however, the UNIZ NBEE (LCD) group exhibited a wider distribution of maximum force values, while the Asiga MAX (DLP) group showed greater variation in elastic modulus values. Regarding resin type, the TA-28 material displayed higher median values and a wider distribution than Clear-A for both parameters. When the thickness factor was evaluated, 1 mm specimens demonstrated higher maximum force values, whereas their effect on elastic modulus was more limited. With respect to specimen geometry, flat specimens clearly reached the highest maximum force values, while the short dumbbell geometry exhibited the lowest performance. Conversely, the long thin dumbbell geometry was notable for reaching higher elastic modulus values.

When the differences observed in the classifications based on the main factors were statistically examined (Table 2), printer type was found to have no statistically significant effect on maximum force (Kruskal–Wallis, *p* = 0.357, ε^2^ = 0.000). In contrast, resin type (*p* < 0.001, r = 0.766) and thickness (*p* < 0.001, r = 0.831) were found to have significant effects with large effect sizes on maximum force. Specimen shape also exhibited a statistically significant effect on maximum force (Kruskal–Wallis, *p* < 0.001, ε^2^ = 0.533), and this effect was found to be of a high magnitude.

When evaluated in terms of elastic modulus, the effect of printer type was not statistically significant, although it showed a borderline value (*p* = 0.052, ε^2^ = 0.033). In contrast, resin type (*p* < 0.001, r = 0.395) and thickness (*p* = 0.008, r = 0.327) were found to have significant and moderate effects on elastic modulus. The effect of specimen shape on elastic modulus was found to be very high (Kruskal–Wallis, *p* < 0.001, ε^2^ = 0.664).

In the post hoc analyses regarding specimen shape (Table 3) (Dunn’s test with Bonferroni correction), flat specimens were found to exhibit significantly higher values for maximum force compared with all other geometries (*p* < 0.001), and the short dumbbell geometry demonstrated significantly lower performance than all other groups (*p* < 0.01). In contrast, no significant difference was found between the long thin dumbbell and long dumbbell geometries in terms of maximum force (*p* = 0.792).

In the post hoc analyses for elastic modulus, the short dumbbell geometry was found to have significantly lower values than all other groups (*p* < 0.001), and the long thin dumbbell geometry showed significantly higher values than both the flat and short dumbbell groups (*p* < 0.001). While no significant difference was observed between the flat and long dumbbell geometries (*p* = 0.064), a significant difference was found between the long thin dumbbell and long dumbbell geometries (*p* = 0.049).

## 4. Discussion

The aim of this study was to evaluate the effects of specimens produced in different thicknesses, lengths, and geometries, using two different clear aligner materials obtained from three different 3D printers, on tensile strength and modulus of elasticity. The findings demonstrated that the evaluated criteria produced significant differences. Accordingly, the null hypothesis stating that the proposed material and design parameters had no effect on tensile strength and modulus of elasticity was rejected.

In recent years, it has been reported that patients embrace aligner treatment more readily [4]. This situation has caused a substantial increase in aligner production processes and has created a need for an accessible and error free production workflow [10]. At this point, with the development of 3D printing technologies, in-house production has come to the forefront. In in-house production, the accuracy and reproducibility of the planned printing process are essential for effective treatment. Studies in this field have reported that accuracy in 3D printing can be considerably influenced by factors such as the printer technology used, the type of printing, the limitations of low cost devices, resin formulations, and production parameters [13,24].

Although factors related to the production process are critical for the accuracy of 3D printed aligners, the ultimate clinical efficacy is largely determined by the mechanical properties of the material used [20]. Indeed, it has been reported that the mechanical performance of the thermoplastic material used in aligner production, together with aligner thickness and geometric design, plays a decisive role in achieving the desired outcomes [21]. Therefore, differences observed in the mechanical properties of aligner materials can directly affect the biomechanical efficacy and ultimate clinical outcome of treatment [2,21]. Ahn et al. [32], demonstrated that thermoplastic polymers commonly used in orthodontics have limitations such as dimensional instability, low strength, and insufficient wear resistance, but that aligner performance can be significantly improved with appropriate material selection and optimized production methods.

Accordingly, evaluating the combined effects of different printer technologies, resin materials, and design parameters on mechanical performance is of great importance. However, the limited number of studies in which these variables are addressed together constitutes a significant gap in the existing literature. For this reason, the present study examined the effects of these parameters on tensile strength and elastic modulus in vitro. It has been noted in the literature that, due to inter individual and time dependent variability of the oral environment, its interaction with dental materials cannot be fully standardized [33]. This situation increases the importance of in vitro mechanical tests. The elastic modulus measured in tensile tests has been reported to be appropriate for evaluating the force required to produce tooth movement [34]. Indeed, it is also known that the flexibility properties of aligners are inversely proportional to the elastic modulus [35].

In the present study, specimens prepared from different resins were produced using 3D printing technology. The conventional thermoforming production process is associated with numerous geometric inaccuracies and irregularities in aligner thickness [15]. This may lead to uncertainties in aligner performance and treatment outcomes [36]. To overcome these disadvantages, the 3D printing method has been recommended [16]. In the study by Sayahpour et al. [37] in house thermoformed aligners were found to exhibit better mechanical properties than directly printed aligners in terms of wear resistance, hardness, and force degradation. Therefore, in order to evaluate production parameters in a more controlled manner and to more clearly elucidate the effects of material and design variables, specimens in this study were prepared using the 3D printing method.

On the other hand, simplified specimen designs were used in the present study instead of complete aligner geometries. Although these geometries do not fully represent the complex morphology of clinical aligners, they allow the effects of design parameters on mechanical behavior to be evaluated individually under standardized conditions. Real aligners contain regions with varying thicknesses, curvatures, and cross-sectional transitions, all of which may influence stress distribution and force transmission. Therefore, the findings of the present study should be interpreted as demonstrating the fundamental mechanical effects of material and design parameters rather than directly representing the behavior of a complete aligner. Nevertheless, the results provide valuable insight into the mechanical behavior of different regions within clinical aligners. Indeed, studies evaluating mechanical properties have frequently employed simplified specimen geometries similar to those used in the present study in order to test materials under comparable and standardized conditions [6,38,39].

In the present study, the effect of different printer types on mechanical properties was evaluated using three different printers in the 3D printing process. As a result, printer type was found to have no statistically significant effect on maximum force (*p* = 0.357) or elastic modulus (*p* = 0.052). When the mean maximum force values were examined, the values for Clear A obtained with the UNIZ NBEE (LCD), Asiga MAX (DLP), and Ackuretta SOL (LCD) printers were 63.3 N, 47.8 N, and 53.8 N, respectively, while those for TA 28 were 179.9 N, 138.8 N, and 161.3 N, respectively. In terms of elastic modulus, the corresponding values for Clear A were 663.6 N/mm^2^, 311.0 N/mm^2^, and 351.1 N/mm^2^, while those for TA 28 were 639.5 N/mm^2^, 910.9 N/mm^2^, and 555.9 N/mm^2^.

Although differences existed among the mean values for some printer resin combinations, these differences did not alter the overall trend and did not reach statistical significance. This finding indicates that, despite the use of different 3D printing technologies (LCD and DLP), the mechanical properties obtained were generally at similar levels. In particular, although the effect of printer type on elastic modulus was at the borderline of statistical significance (*p* = 0.052), it was not significant, and the low effect size (ε^2^ = 0.033) indicates that the contribution of this parameter to mechanical performance is limited.

Nevertheless, different findings have been reported in the literature. Zinelis et al. [40] identified significant differences among printers despite using the same resin and the same post curing protocol in all groups, and noted that this could be attributed to parameters specific to the printing process. They emphasized that factors such as light intensity, exposure time, and curing depth can directly influence the mechanical properties of the three-dimensional structure. The researchers also reported that LCD printers can yield higher mechanical properties, such as elastic modulus and hardness, than DLP printers [40,41].

In contrast, in the present study, printer type was found to have no statistically significant effect on either maximum force or elastic modulus (*p* > 0.05). This finding is consistent with the results of Bhardwaj et al. [42] who reported that different 3D printer technologies (LCD and DLP) provide similar mechanical properties. In their study, Bhardwaj et al. [42] reported no significant differences among various commercial printers in terms of Martens hardness, indentation modulus, and elastic index. Miglioratti et al. [39] compared the mechanical properties of specimens produced using the Phrozen Sonic XL 4K and AccuFab L4D printers. The Phrozen printer yielded a higher tensile elastic modulus (1131.30 ± 63.99 MPa), whereas the AccuFab printer demonstrated higher ultimate tensile strength (23.99 ± 1.16 MPa) and elongation at break (75.76 ± 27.86%). However, the authors reported that these differences were not statistically significant. In this respect, the findings of the present study are in agreement with their results.

Although Zinelis et al. [40] reported significant differences among printers, the fact that the differences in our study did not reach statistical significance suggests that standardization of production parameters and post polymerization processes may have minimized the variations potentially arising from printer technology. This supports the notion that printer type alone may not be a determining factor and that mechanical performance is more strongly influenced by material and design parameters.

In the present study, two different resins (TA 28 and Clear A) were used to determine the effect of resin type on mechanical properties. Resin type was found to have a statistically significant effect on both maximum force (*p* < 0.001, r = 0.766) and elastic modulus (*p* < 0.001, r = 0.395). This finding clearly demonstrates the determining role of material composition on mechanical performance. In particular, the higher maximum force and elastic modulus values exhibited by TA 28 compared with Clear A may be related to the chemical structure and crosslinking density of the resin used. The high effect size obtained for maximum force (r = 0.766) indicates that the influence of resin type on mechanical strength is quite strong. Although the effect size obtained for elastic modulus was lower (r = 0.395), it nevertheless points to a significant effect.

A review of the literature reveals that the type of aligner resin used produces significant differences in both thermoformed aligners and aligners produced by 3D printing. Shirey et al. [20] tested the mechanical properties of specimens prepared from two thermoforming materials, EX30 and LD30 (Align Technology Inc., San Jose, CA, USA), and two direct 3D printing resins, Material X (EnvisionTEC, Inc., Dearborn, MI, USA) and OD Clear TF. They reported that mechanical properties varied according to the material used, and that the elastic modulus of Material X was significantly higher than that of EX30, LD30, and OD Clear TF [20]. Another study reported that the elastic modulus of 3D printed groups was consistent with that of Invisalign, [43] and was higher than that of clear aligners produced by the thermoforming method [2]. Ryu et al. [44] performed tensile tests after thermoforming to evaluate the durability of different thermoplastic materials and reported that elastic modulus values varied depending on the material type. In another study using different aligner materials, the hardness and elastic modulus values of Duran and Erkodur materials were reported to be approximately twice as high as those of Hardcast, and these materials were noted to provide more clinically effective tooth movements [21]. Migliorati et al. [39] investigated the effects of various factors, including printer type, resin type, layer height, and specimen thickness, on mechanical properties and reported that resin type was the variable responsible for the most pronounced and statistically significant differences among the parameters evaluated. These findings indicate that different production methods and material compositions are decisive factors for mechanical properties. In the present study, the higher maximum force and elastic modulus values of TA 28 compared with Clear A are consistent with this trend reported in the literature. The superior strength and rigidity of the TA-28 resin make it a suitable material for aligners requiring greater stiffness and mechanical durability. In contrast, the higher ductility and shape-memory properties of Clear-A resin highlight its potential for applications where flexibility and adaptability are prioritized, potentially enhancing patient comfort and improving performance in clinical situations involving complex dental geometries [45]. In conclusion, both the findings of the present study and the data in the literature indicate that resin type plays a determining role in the mechanical performance of aligner materials. This demonstrates that taking chemical structure and mechanical properties into account during material selection is critical for treatment efficacy.

Another factor evaluated in the present study was specimen thickness. Thickness was found to have a statistically significant effect on both maximum force (*p* < 0.001, r = 0.831) and elastic modulus (*p* = 0.008, r = 0.327). The very high effect size obtained for maximum force (r = 0.831) indicates that thickness is one of the most decisive parameters for mechanical strength. Similarly, a significant effect on elastic modulus was also observed. Consistent with the findings of the present study, Migliorati et al. [39] reported that 2 mm thick specimens (41.46 ± 1.89 MPa) exhibited significantly higher ultimate tensile strength than 1 mm thick (26.62 ± 1.36 MPa) and 0.5 mm thick (25.22 ± 6.43 MPa) specimens. Similarly, Khalil et al. [38] found that aligner specimens with a thickness of 0.7 mm demonstrated significantly greater flexural strength compared with specimens having a thickness of 0.5 mm.

The marked increase in maximum force values with increasing thickness can be explained by the greater resistance of thicker specimens under load. This is related to the fact that an increase in cross sectional area alters stress distribution and enables the material to bear greater loads before fracture. The increase in elastic modulus with increasing thickness indicates that the rigidity of the material increases and, consequently, its flexibility decreases. Because there is an inverse relationship between elastic modulus and flexibility, it can be stated that thicker specimens are more resistant to deformation but behave less flexibly. From an orthodontic perspective, this suggests that, although thicker aligners have the potential to generate higher forces, they may make it more difficult for tooth movement to occur in a controlled manner within physiological limits. In contrast, thinner materials, owing to their lower elastic modulus, may behave more flexibly and contribute to a more balanced transmission of forces. Indeed, it is known that the orthodontic forces produced by clear aligners are directly dependent on material thickness and the mechanical properties of the thermoplastic material used [2,21,46]. However, in contrast to the results of our study, Ryu et al. [44] reported that, in some conventional heat shaped thermoplastic materials (Duran, Essix ACE), the elastic modulus decreased as thickness increased. This may stem from material strain and changes in molecular alignment occurring during the thermoplastic forming process. In contrast, in our study, the use of 3D printing technology and the increase in rigidity of the layered structure with increasing thickness are considered the main reasons for the increase in elastic modulus values. This finding reveals that the mechanical behavior of 3D printing resins differs from that of conventional thermoplastic materials. In this context, evaluating aligner thickness not merely as a general design parameter but as a dynamic property that can be optimized regionally becomes important.

Indeed, the ability to adjust aligner thickness for different tooth movements and anatomical regions adds a new dimension to conventional aligner therapy. Thanks to the 3D printing technologies developed in recent years, it has been reported that variable thickness designs can be incorporated within a single aligner, thereby enabling different force levels to be obtained in different regions [47]. This approach not only enhances force control but also enables the integration of biomechanical principles employed in functional orthodontic appliances into aligner systems. Therefore, considering the marked effect of thickness on mechanical properties observed in the present study, it is anticipated that personalized and regionally optimized aligner designs may enhance treatment efficacy in the future.

In this study, specimen geometry was found to have a statistically significant and large effect on both maximum force (*p* < 0.001, ε^2^ = 0.533) and elastic modulus (*p* < 0.001, ε^2^ = 0.664). This finding indicates that specimen design can be as decisive as the material itself in determining mechanical performance. In terms of maximum force, flat specimens exhibited significantly higher values than all dumbbell geometries. In contrast, the short dumbbell geometry showed the lowest maximum force values. This suggests that the cross sectional narrowing in dumbbell geometries and the concentration of stress in specific regions may negatively affect fracture behavior. The fact that this stress concentration was more pronounced, particularly in the short dumbbell geometry, may have resulted in lower strength values. When geometry-related differences were evaluated in terms of elastic modulus, specimen design was found to have a substantial influence on the stiffness behavior of the material. An increase in elastic modulus indicates greater resistance to deformation and, consequently, increased rigidity. From this perspective, geometric design appears to play an important role in mechanical behavior. When the dumbbell groups were evaluated separately, the short dumbbell geometry exhibited the lowest elastic modulus values, which may be related to increased stress concentrations and localized deformation within the shorter gauge region. In contrast, the wider distribution of load across the longer gauge region in the long dumbbell geometry may have resulted in a more homogeneous elastic deformation behavior. Interestingly, the long thin dumbbell group exhibited higher elastic modulus values than the long dumbbell group. This difference may be attributed to variations in stress distribution associated with changes in cross-sectional area. However, since stress distribution was not directly analyzed in the present study, further investigations, such as finite element analyses, are required to confirm this mechanism.

When these findings are evaluated from a clinical perspective, since aligners are produced patient specifically to conform to dental morphology, there is no standard specimen geometry. Nevertheless, the results obtained indicate that cross-sectional changes and local narrowings can significantly influence mechanical behavior. The low force values observed in the dumbbell geometry, particularly in the short dumbbell, suggest that stress concentration may increase in narrowed cross sections. This indicates that locally thinned areas of the aligner, such as interproximal regions, may behave more weakly mechanically and that deformation under load may begin in these regions. In contrast, the higher force values exhibited by flat geometries indicate that regions in contact with the broad buccal and lingual surfaces of the teeth play a more effective role in force transmission. These findings further support the importance of region-specific thickness modifications in aligner design. In this regard, Grant et al. [47] reported that strategically increasing the labiolingual thickness of directly printed aligners may reduce undesirable side effects during tooth movement and enable more controlled tipping movements, thereby resulting in more predictable biomechanical outcomes through localized thickness adjustments.

In addition, the present findings suggest that regional modifications in aligner thickness and geometry may be used to optimize force delivery. The significant increase in maximum force observed with increasing specimen thickness, together with the superior force-bearing capacity of flat geometries, indicates that thicker aligner regions and broader surface areas may provide more efficient force transmission. Therefore, in clinical situations requiring greater orthodontic loading, selective increases in aligner thickness or the incorporation of broader and flatter contact surfaces may enhance force delivery. Conversely, thinner regions may be advantageous where lower force levels are desired. Such region-specific design modifications could allow a more controlled distribution of orthodontic forces within a single aligner.

This study has several main limitations. First, due to the in vitro design of the research, the long-term effects of dynamic factors in the oral environment, such as salivary enzymes, pH variations, and thermal cycling, on the materials could not be fully reflected. In addition, the flat and dumbbell shaped specimens used to standardize the mechanical tests may differ from the complex three-dimensional anatomical form of actual clear aligners and from the stress distribution that this form creates. Furthermore, advanced clinical studies are needed to relate the differences in mechanical properties obtained to clinical outcomes. In this regard, evaluating the time dependent mechanical properties of materials produced by 3D printing through both experimental and clinical studies is important for a better understanding and optimization of the clinical effectiveness of these systems.

## 5. Conclusions

Within the limitations of this in vitro study, resin type (TA 28 or Clear A), specimen thickness, and geometry were found to have significant effects on both maximum force and elastic modulus, while printer type (LCD or DLP) had no significant effect on mechanical performance. Among the parameters evaluated, thickness and resin type were identified as the most important factors determining mechanical behavior.

From a clinical perspective, these findings indicate that, in order to achieve a successful biomechanical outcome in in-house production processes, clinicians should focus on the mechanical characteristics of the resin to be used and on aligner design parameters rather than on device selection.

## Figures and Tables

**Table 1:** Distribution of samples used in the study.

Printer	Resin	Thickness	Lenght	Shape
Ackuretta SOL (Ackuretta, Taipei, Taiwan)	Clear A	0.5 mm	Long	Dumbell
			Short	Flat
	Graphy	0.5 mm	Long	Dumbell
		1 mm	Short	Flat
Asiga MAX (Asiga, Sydney, Australia)	Clear A	0.5 mm	Long	Dumbell
			Short	Flat
	Graphy	0.5 mm	Long	Dumbell
		1 mm	Short	Flat
Uniz NBEE (UNIZ Technology, China)	Clear A	0.5 mm	Long	Dumbell
			Short	Flat
	Graphy	0.5 mm	Long	Dumbell
		1 mm	Short	Flat

**Table 2:** Statistical comparison of maximum force and elastic modulus according to experimental factors.

Factor	Test	Maximum Force		Elastic Modulus	
		*p*	Effect Size	*p*	Effect Size
Printer Type	Kruskal–Wallis	0.357	0.000	0.052	0.033
Resin Type	Mann–Whitney U	0.000 ***	0.766	0.000 ***	0.395
Thickness (mm)	Mann–Whitney U	0.000 ***	0.831	0.008 **	0.327
Shape	Kruskal–Wallis	0.000 ***	0.533	0.000 ***	0.664

** *p* < 0.01, *** *p* < 0.001.

**Table 3:** Post hoc pairwise comparisons of specimen geometries for maximum force and elastic modulus (Dunn test with Bonferroni correction).

Comparison	Maximum Force		Elastic Modulus	
	Z	*p*	Z	*p*
Flat-Long Dumbbell	4.669	0.000 ***	−1.854	0.064
Flat-Long Thin Dumbbell	3.411	0.001 **	−3.555	0.000 ***
Long Dumbbell-Long Thin Dumbbell	−0.264	0.792	−1.970	0.049 *
Flat-Short Dumbbell	7.848	0.000 ***	5.940	0.000 ***
Long Dumbbell-Short Dumbbell	2.902	0.004 **	7.115	0.000 ***
Long Thin Dumbbell-Short Dumbbell	2.633	0.008 **	7.779	0.000 ***

Note: Dunn post hoc test with Bonferroni correction was applied for multiple comparisons. Values are presented as Z statistics and corresponding *p*-values. * *p* < 0.05, ** *p* < 0.01, *** *p* < 0.001.

## Data Availability

The original contributions presented in the study are included in the article; further inquiries can be directed to the corresponding author.

## Supplementary Materials

The following supporting information can be downloaded at: https://www.mdpi.com/article/10.3390/polym18121486/s1, Figure S1. Heatmap representation of maximum force (N) across combined printer type, resin type, and specimen geometry groups. Figure S2. Heatmap representation of elastic modulus (N/mm^2^) across combined printer type, resin type, and specimen geometry groups. Table S1. Descriptive statistics-Maximum Force (N). Table S2. Descriptive statistics-Elastic Modulus (N/mm^2^).

## Abbreviations

3D	Three-dimensional
DLP	Digital Light Processing
LCD	Liquid Crystal Display
ICC	Intraclass Correlation Coefficient
ISO	International Organization for Standardization
