# Supplementary material for: Influence of 3D Printer Type, Resin Material, Thickness, and Geometry on the Mechanical Properties of Directly Printed Clear Aligners
Source: Polymers (Basel). 2026 Jun 13;18(12):1486. doi: 10.3390/polym18121486 (PMC13307045; doi:10.3390/polym18121486)
Supplement: Supplementary file 1 [file polymers-18-01486-s001.zip › polymers-4342148-supplementary.pdf]

Suplementary Tables

Suplementary Table 1. Descriptive statistics - Maximum Force (N)

Printer	Resin	Thickness	Shape	n	Mean	SD	Median	Min	Max
Ackuretta SOL (LCD)	Clear-A	0.5	Flat	5	119.901	6.607	120.898	110.944	128.868
Ackuretta SOL (LCD)	Clear-A	0.5	Short Dumbbell	5	14.921	6.499	10.381	10.104	23.702
Ackuretta SOL (LCD)	Clear-A	0.5	Long Dumbbell	5	26.658	8.733	24.211	17.363	40.857
Ackuretta SOL (LCD)	TA-28	0.5	Flat	5	236.299	15.883	241.365	218.446	257.136
Ackuretta SOL (LCD)	TA-28	0.5	Short Dumbbell	5	51.139	5.261	50.123	45.567	59.430
Ackuretta SOL (LCD)	TA-28	0.5	Long Thin Dumbbell	5	70.153	2.933	70.153	66.740	74.665
Ackuretta SOL (LCD)	TA-28	1	Flat	5	292.889	19.281	296.842	261.884	313.581
Ackuretta SOL (LCD)	TA-28	1	Long Dumbbell	5	156.177	35.371	175.152	94.933	177.787
Asiga MAX (DLP)	Clear-A	0.5	Flat	5	84.182	5.396	84.182	78.042	92.001
Asiga MAX (DLP)	Clear-A	0.5	Short Dumbbell	5	24.193	1.499	23.681	22.531	26.558
Asiga MAX (DLP)	Clear-A	0.5	Long Dumbbell	5	35.006	2.408	33.789	33.390	39.169
Asiga MAX (DLP)	TA-28	0.5	Flat	5	158.705	19.261	158.705	134.244	185.561
Asiga MAX (DLP)	TA-28	0.5	Short Dumbbell	5	82.919	3.843	83.456	78.836	87.943
Asiga MAX (DLP)	TA-28	0.5	Long Thin Dumbbell	5	85.380	7.119	82.323	79.142	96.377
Asiga MAX (DLP)	TA-28	1	Flat	5	236.728	16.590	234.140	220.617	263.152
Asiga MAX (DLP)	TA-28	1	Long Dumbbell	5	130.087	7.593	128.682	123.070	140.438
UNIZ NBEE (LCD)	Clear-A	0.5	Flat	5	99.669	10.001	99.669	89.688	112.637
UNIZ NBEE (LCD)	Clear-A	0.5	Short Dumbbell	5	38.250	1.993	37.831	36.197	41.541
UNIZ NBEE (LCD)	Clear-A	0.5	Long Dumbbell	5	51.920	3.917	51.920	46.576	56.820
UNIZ NBEE (LCD)	TA-28	0.5	Flat	5	229.418	12.200	232.391	212.755	244.241
UNIZ NBEE (LCD)	TA-28	0.5	Short Dumbbell	5	67.671	8.897	68.383	55.604	77.391
UNIZ NBEE (LCD)	TA-28	0.5	Long Thin Dumbbell	5	112.472	15.133	117.753	91.583	128.975
UNIZ NBEE (LCD)	TA-28	1	Flat	5	300.432	33.339	295.567	258.735	350.567
UNIZ NBEE (LCD)	TA-28	1	Long Dumbbell	5	189.469	9.627	190.341	177.759	203.635

SD: Standard Deviation. Values are presented as mean ± SD.

Suplementary Table 2. Descriptive statistics - Elastic Modulus (N/mm²)

Printer	Resin	Thickness	Shape	n	Mean	SD	Median	Min	Max
Ackuretta SOL (LCD)	Clear-A	0.5	Flat	5	427.589	30.008	434.340	394.299	459.639
Ackuretta SOL (LCD)	Clear-A	0.5	Short Dumbbell	5	30.467	16.737	28.124	12.748	57.339
Ackuretta SOL (LCD)	Clear-A	0.5	Long Dumbbell	5	595.180	69.035	603.560	507.390	694.499
Ackuretta SOL (LCD)	TA-28	0.5	Flat	5	705.723	35.345	714.603	660.840	753.416
Ackuretta SOL (LCD)	TA-28	0.5	Short Dumbbell	5	30.467	16.737	28.124	12.748	57.339
Ackuretta SOL (LCD)	TA-28	0.5	Long Thin Dumbbell	5	867.273	41.688	867.273	807.785	906.487
Ackuretta SOL (LCD)	TA-28	1	Flat	5	603.647	98.042	626.240	436.566	691.766
Ackuretta SOL (LCD)	TA-28	1	Long Dumbbell	5	572.155	145.919	572.155	387.183	722.416
Asiga MAX (DLP)	Clear-A	0.5	Flat	5	341.530	90.999	341.530	243.593	441.042
Asiga MAX (DLP)	Clear-A	0.5	Short Dumbbell	5	19.362	7.283	17.038	10.725	29.733
Asiga MAX (DLP)	Clear-A	0.5	Long Dumbbell	5	572.152	80.720	579.468	442.696	663.151
Asiga MAX (DLP)	TA-28	0.5	Flat	5	969.232	31.720	953.582	949.286	1,024.100
Asiga MAX (DLP)	TA-28	0.5	Short Dumbbell	5	9.350	1.374	9.427	7.828	11.498
Asiga MAX (DLP)	TA-28	0.5	Long Thin Dumbbell	5	1,501.574	223.981	1,447.290	1,290.700	1,827.980
Asiga MAX (DLP)	TA-28	1	Flat	5	825.212	44.641	831.715	774.965	888.067
Asiga MAX (DLP)	TA-28	1	Long Dumbbell	5	1,249.028	157.029	1,261.170	1,083.630	1,468.880
UNIZ NBEE (LCD)	Clear-A	0.5	Flat	5	709.304	8.496	709.304	700.737	721.104
UNIZ NBEE (LCD)	Clear-A	0.5	Short Dumbbell	5	122.111	43.891	122.111	65.127	169.939
UNIZ NBEE (LCD)	Clear-A	0.5	Long Dumbbell	5	1,159.310	61.444	1,159.310	1,070.230	1,227.280
UNIZ NBEE (LCD)	TA-28	0.5	Flat	5	686.506	68.791	702.560	590.985	774.737
UNIZ NBEE (LCD)	TA-28	0.5	Short Dumbbell	5	185.968	48.710	164.483	149.033	270.586
UNIZ NBEE (LCD)	TA-28	0.5	Long Thin Dumbbell	5	915.301	51.714	901.046	867.796	1,002.440
UNIZ NBEE (LCD)	TA-28	1	Flat	5	545.825	47.947	550.045	469.901	593.065
UNIZ NBEE (LCD)	TA-28	1	Long Dumbbell	5	864.088	83.578	898.145	763.471	958.327

SD: Standard Deviation. Values are presented as mean ± SD.

Suplementary Figures

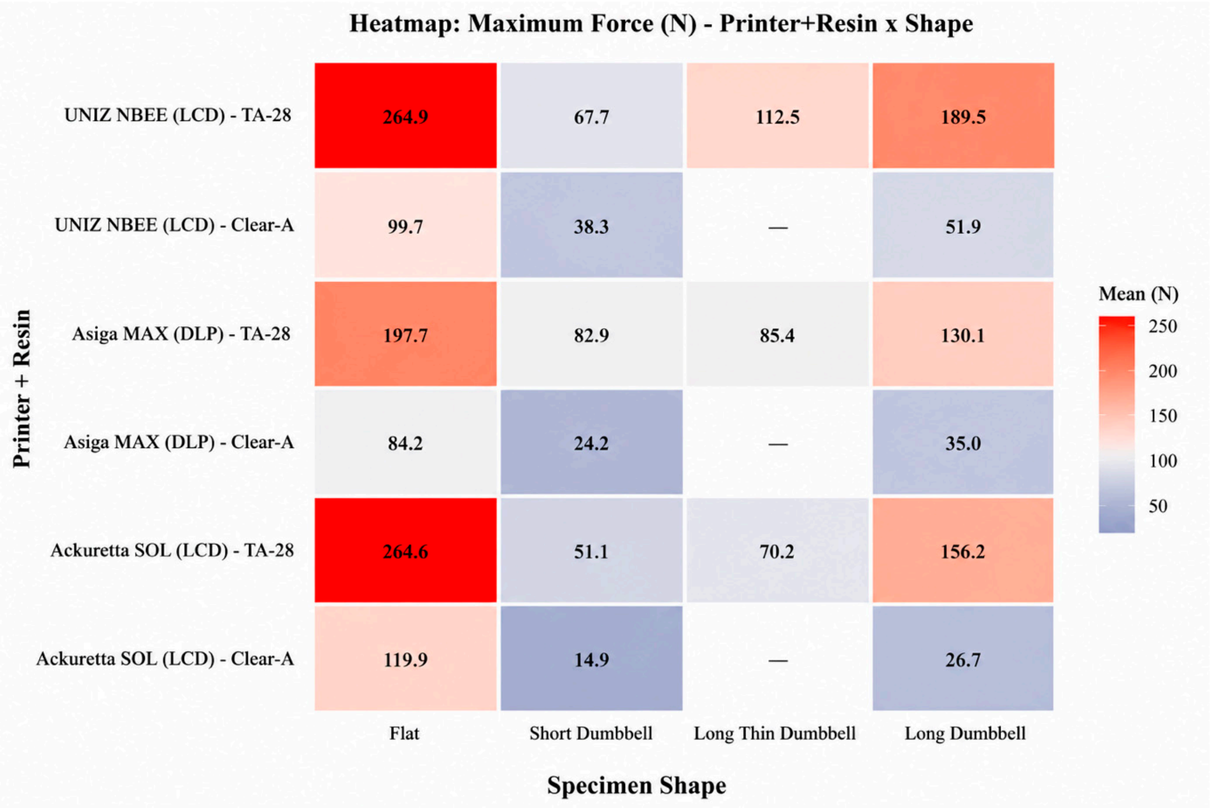

Suplementary Figure 1. Heatmap representation of maximum force (N) across combined printer type, resin type, and specimen geometry groups.

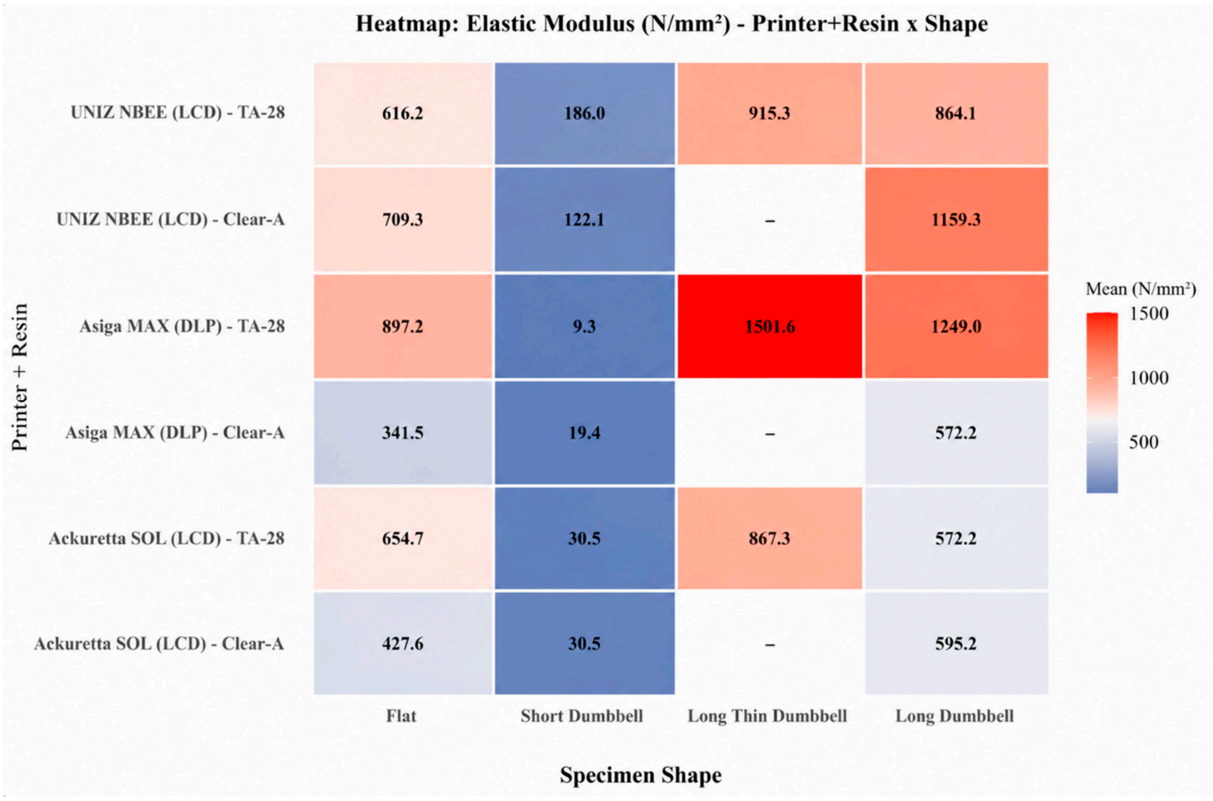

Suplementary Figure 2. Heatmap representation of elastic modulus (N/mm²) across combined printer type, resin type, and specimen geometry groups.
